# Serum Magnesium Concentrations in the United States—An Updated Population Reference Interval in Children and Adults

**DOI:** 10.1016/j.tjnut.2026.101539

**Published:** 2026-04-16

**Authors:** Keyi Jiao, Rebecca Costello, Jaime Gahche, Andrea Rosanoff, Taylor C Wallace

**Affiliations:** 1 Department of International Health, Johns Hopkins Bloomberg School of Public Health, Baltimore, MD, United States; 2 Center for Magnesium Education and Research, Pahoa, HI, United States; 3 Office of Dietary Supplements, United States NIH, Bethesda, MD, United States; 4 Gerald J. and Dorothy R. Friedman School of Nutrition Science and Policy, Tufts University, Boston, MA, United States; 5 School of Medicine and Health Sciences, George Washington University, Washington, DC, United States; 6 Think Healthy Group, LLC, Washington, DC, United States

**Keywords:** magnesium, hypertension, diabetes, nutritional status, reference value, magnesium deficiency

## Abstract

**Background:**

Serum magnesium is a practical biomarker for assessing nutritional status in clinical settings, yet reference intervals commonly used in the United States largely reflect data from NHANES I (1971–1974).

**Objectives:**

The aim of this study is to describe serum magnesium concentrations across the US population and establish contemporary population-based reference intervals for children and adults.

**Methods:**

This cross-sectional study used data from nonpregnant and nonlactating civilian participants in the 2021–2023 NHANES data cycles. Reference intervals were estimated following the International Federation of Clinical Chemistry recommendations. Primary analyses categorized children (aged 12–18 y) by sex and adults (aged ≥19 y) by sex, age, and metabolic health status [total population, metabolically healthy, hypertension, diabetes, and chronic kidney disease (CKD)]. Linear regression models were used to compare subgroups, and sensitivity analyses were conducted to assess robustness of primary findings.

**Results:**

The final analytic sample included 787 children and 5474 adults. Girls had significantly lower mean serum magnesium than boys (*P* = 0.003), although differences were small. The reference interval was 1.70–2.19 mg/dL (0.70–0.90 mmol/L; 1.40–1.80 mEq/L) for boys and 1.64–2.18 mg/dL (0.68–0.90 mmol/L; 1.35–1.79 mEq/L) for girls based on data from the total population. Mean serum magnesium concentrations were significantly lower in adult males and females with diabetes (*P* < 0.001; *P* < 0.001), hypertension (*P* = 0.012; *P* = 0.029), or CKD (*P* = 0.007; *P* = 0.002) compared with those who were metabolically healthy, respectively, and in females compared with males (*P* < 0.001). The reference interval was 1.72–2.26 mg/dL (0.71–0.93 mmol/L; 1.42–1.86 mEq/L) for males and 1.70–2.21 mg/dL (0.70–0.91 mmol/L; 1.40–1.82 mEq/L) for females based on data from the metabolically healthy population. Estimated prevalence of chronic latent magnesium deficiency (CLMD), represented by a serum magnesium concentration <2.06 mg/dL (0.85 mmol/L; 1.70 mEq/L), was 67.8% in adults.

**Conclusions:**

This study provides contemporary population-based reference intervals for serum magnesium for children and adults and suggests that a substantial portion of the US population is at risk of CLMD.

## Introduction

Magnesium is an essential mineral and the preeminent divalent intracellular cation within human cells, central to numerous biological processes such as, but not limited to, oxidative phosphorylation, energy production, glycolysis, potassium and calcium transport, and the synthesis of proteins and nucleic acids, among others [1,2]. Enzymatic databases list magnesium as an activator or cofactor for >200 and >600 enzymes in the human body, respectively [[3], [4], [5]].

Magnesium homeostasis is tightly regulated by hormonal and nonhormonal mechanisms to maintain optimal intracellular and extracellular levels. The kidneys serve as the main regulator of magnesium homeostasis, where magnesium is filtered by the glomeruli and reabsorbed or excreted in the urine [1,2,6]. Parathyroid hormone increases renal magnesium reabsorption in response to low serum magnesium concentrations, whereas calcitriol enhances intestinal absorption by upregulating the expression of transient receptor potential melastatin 6 in the small intestine [1,6,7]. Approximately 30%–50% of dietary magnesium is absorbed by the body; however, when intake is low, absorption can increase to ∼80% [8,9]. Although magnesium can be stored in muscle fibers, where it plays an important role in the regulation of muscle contraction by antagonizing the action of calcium, bone serves as the largest magnesium reservoir in the body, where it also contributes to the density and strength of the skeleton. Bone surface magnesium concentrations (∼30%) are closely related to serum magnesium concentrations [10], indicating the continuous exchange between bone and blood. Despite renal conservation, magnesium can be pulled from bone (as well as from muscle tissue and internal organs) to maintain normal serum magnesium concentrations when dietary intake is low [1,2,6,11]. Certain medications (e.g., diuretics, aminoglycosides, proton pump inhibitors, cetuximab, and immunosuppressants) can also cause serum magnesium concentrations to decrease through renal magnesium wasting [2,6].

A substantial body of evidence has linked low serum magnesium concentrations with several common metabolic conditions, including but not limited to hypertension [[12], [13], [14]], type 2 diabetes [[15], [16], [17], [18]], and chronic kidney disease (CKD) [19], among others. Low serum magnesium status is also associated with increased mortality risk and accelerated disease progression among individuals with the aforementioned metabolic conditions [12,[20], [21], [22], [23]]. Although clinical manifestations of magnesium deficiency are thought to be relatively uncommon, chronic latent magnesium deficiency (CLMD), a condition in which serum magnesium concentrations may remain within conventionally defined reference intervals despite progressive depletion of magnesium stores, is a common occurrence that is higher among older adults [[24], [25], [26]]. CLMD is generally indicated by a serum magnesium concentration <2.06 mg/dL (0.85 mmol/L; 1.70 mEq/L) [2,[27], [28], [29]]. Abnormalities in serum magnesium have been suggested to be one of the most underdiagnosed serum electrolyte disturbances in clinical practice [30]. Approximately half of the US population consumes less than the Estimated Average Requirement for magnesium, and some age groups consume substantially less [31,32]. Oral magnesium supplementation has been consistently shown to raise serum magnesium concentrations [14,33] and both dietary and supplemental magnesium interventions have demonstrated promising preventive and therapeutic effects across these conditions [13,21,[34], [35], [36], [37]].

Accurate assessment of magnesium status and identification of subacute or CLMD remains a significant clinical challenge. Because magnesium is an intracellular cation also predominantly (>99%) stored in bone, muscle, and soft tissues, the diagnostic value of routine blood tests is somewhat limited in the ability of these tests to detect deficiency or inadequacy. The most reliable method for assessing nutritional status is the magnesium load (tolerance/retention) test, which involves measuring 24-h retention and excretion of intravenously administered magnesium [1,2,38]. However, this test is invasive, cumbersome, nonroutine, and potentially risky in individuals with renal impairment. Thus, there is a fairly broad scientific consensus that serum magnesium concentrations represent the most practical clinical approach for evaluating and monitoring magnesium status in the healthcare setting [27,31].

Despite the clinical relevance of magnesium, serum magnesium concentrations are not routinely included or reported in standard metabolic panels used across the healthcare setting. Furthermore, there is no universally accepted reference interval for serum magnesium that defines metabolic health [31]. Multiple reference intervals are currently used in both clinical and research settings in the United States and internationally [27]. At least 4 distinct reviews or consensus manuscripts have proposed serum magnesium reference intervals in the scientific literature over the last decade [27,29,39,40]. Compounding this issue, the reference interval most frequently applied (i.e., 1.82–2.32 mg/dL; 0.75–0.96 mmol/L; 1.50–1.91 mEq/L) within many hospital systems in the United States was derived from data collected within the NHANES I (1971–1974) data >50 y ago [41].

The disparate serum magnesium reference intervals in use yield markedly different prevalence estimates of hypomagnesemia in both the clinical and research settings [27,42]. For this reason, the NIH Office of Dietary Supplements collaborated with the Centers for Disease Control and Prevention National Center for Health Statistics (NCHS) to measure serum magnesium concentrations among participants aged ≥12 y in the 2021–2023 data cycles of the nationally representative NHANES. Thus, the objective of this study was to describe serum magnesium concentrations across the US population and to establish a contemporary population-based reference interval for children and adults using the 2021–2023 NHANES data cycles and recommended procedures for estimating reference intervals from the International Federation of Clinical Chemistry and Laboratory Medicine (IFCC), for which this dataset is most appropriate. A forthcoming analysis will explore the cross-sectional relationship between serum magnesium concentrations and cardiometabolic risk factors among NHANES participants to support the design of future interventional studies that seek to establish evidence-based reference intervals.

## Methods

This cross-sectional cohort study is reported per the STROBE guideline [43]. The authors developed and did not deviate from a prespecified statistical analysis plan (Supplementary Material) before downloading the raw data from the NCHS website and undertaking the analyses.

### Study population

NHANES is a series of cross-sectional surveys designed to assess the health and nutritional status of the noninstitutionalized civilian population in the United States. Data are collected in 2-y data cycles using a nationally representative, complex, multistage probability sample, with data gathered through interviews, standardized physical examinations, and laboratory testing. Details on the study design, protocols, and data collection procedures have been described elsewhere [44]. All NHANES protocols were approved by the NCHS Research Ethics Review Board. Written informed consent was obtained from adults (aged ≥18 y). Written informed consent was obtained from the parent or guardian of participants who were children (aged <18 y), with additional assent attained from the children.

Data were from the August 2021 to August 2023 NHANES data cycles, which assessed serum magnesium status among all individuals aged 12+ y as part of the standard biochemistry profile. This data cycle represents the first time that serum magnesium status has been measured among NHANES participants since the NHANES I (1971–1974), which was used to establish current reference intervals (e.g., 0.75–0.95 mmol/L in adults). The initial sample in our study included 11,933 US participants. Participants were excluded if they lacked serum magnesium measurements (*n* = 5609) or were pregnant (*n* = 37) and/or lactating (*n* = 26). This yielded a final analytical sample of 787 children (aged 12–18 y) and 5474 adults (aged ≥19 y) (*n* = 6261) (Supplementary Figure 1). Nationally representative cross-sectional data are widely regarded as the reference standard for establishing population-based reference intervals because they provide a large, diverse sample that reflects the nation’s demographic composition, thereby yielding intervals with high generalizability across the population [45].

Adult participants were categorized by sex (male or female) and metabolic health status (metabolically healthy, hypertension, diabetes, or CKD) in both primary and secondary analyses. Metabolically healthy adults were defined as participants who had optimal concentrations of all metabolic syndrome risk factor variables and were not taking any medications related to these conditions. These variables included *1*) systolic blood pressure (SBP) <120 mm Hg and diastolic blood pressure (DBP) <80 mm Hg; *2*) fasting plasma glucose (FPG) <100 mg/dL (<5.6 mmol/L) and hemoglobin A1c (HbA1c) <5.7%; *3*) triglycerides <150 mg/dL, HDL cholesterol ≥40 mg/dL in males and ≥50 mg/dL in females, and waist circumference <102 cm for males and <88 cm for females (<90 cm for Asian males and <80 cm for Asian females); and *4*) no self-reported use of medications for hyperlipidemia, hypertension, or diabetes [46]. Adults were classified as having type 2 diabetes if they reported a physician diagnosis, reported use of antidiabetic medications, or had FPG ≥126 mg/dL (≥7.0 mmol/L) or HbA1c ≥6.5% [47]. Adult hypertension was defined by a self-reported physician diagnosis, use of antihypertensive medication, or measured SBP ≥130 mm Hg or DBP ≥85 mm Hg [48]. Adult CKD was defined as an estimated glomerular filtration rate <60 mL/min/1.73 m^2^ or an albumin-to-creatinine ratio >30 mg/g [49].

Children were not categorized beyond sex (male or female) in primary analyses because of limitations in characterizing optimal metabolic health. In sensitivity analyses of children, health status was classified as healthy or unhealthy (secondary analyses). Healthy children were defined as those meeting all of the following criteria from the NHANES medical condition questionnaires: no history of asthma and no evidence of liver-related conditions [including viral hepatitis (A, B, or C)], autoimmune liver diseases (e.g., primary biliary cirrhosis, autoimmune hepatitis, or sclerosing cholangitis), genetic liver disorders (e.g., α-1-antitrypsin deficiency, hemochromatosis, or Wilson’s disease), drug- or medication-induced liver injury, alcoholic liver disease, nonalcoholic fatty liver disease or fatty liver disease, liver cancer, liver cysts or abscess, liver fibrosis, or liver cirrhosis [50]. Children with missing values for determining health status were included in the healthy group.

### Assessment of serum magnesium concentrations

Serum magnesium was measured in blood samples collected in NHANES Mobile Examination Centers (MECs) via venipuncture by trained phlebotomists using standardized procedures. Participants were not required to fast before providing blood samples used to assess serum magnesium concentrations [51,52]. After collection, blood was processed in the MEC field laboratory, where samples were centrifuged, and serum was aliquoted into labeled vials, refrigerated or frozen, and shipped under cold-chain conditions to a central reference laboratory. There, serum magnesium concentration was measured by the colorimetric method using a Cobas 8000 analyzer (Roche). This method is based on the reaction of magnesium with xylidyl blue in an alkaline solution containing ethylene glycol tetraacetic acid to mask the calcium in the sample. In alkaline solution, magnesium forms a purple complex with xylidyl blue. The magnesium concentration is measured photometrically via the decrease in the xylidyl blue absorbance at 600 nm. Additional details are provided in the NHANES Laboratory Procedures Manual [52].

Reference intervals for serum magnesium were determined following recommendations of the IFCC [53,54]. We also adhered to the Clinical Laboratory Standards Institute (CLSI) guideline, which recommends a minimum of 120 qualified reference individuals for developing reference interval values [55]. Serum magnesium concentrations (in mg/dL) were analyzed as a continuous variable and reported as the mean ± SE, and the 2.5th and 97.5th percentiles. Males and females were evaluated independently. In adults, linear regression models were used to assess differences in serum magnesium by sex (male or female), age (19–30, 31–50, 51–70, or ≥71 y, to coincide with dietary reference intake life-stage groups), and health status (total population, metabolically healthy, hypertension, diabetes, or CKD). The metabolically healthy group was used as the reference category. Adults with missing metabolic syndrome risk factor variables were excluded from the primary analyses. Sensitivity analyses were conducted in adults by including those with missing metabolic syndrome risk factor variables in the metabolically healthy group, so long as they met ≥3 of the criteria (secondary analyses). For children, linear regression models were used only to assess differences by sex (male or female) (primary analyses). Sensitivity analyses compared healthy and unhealthy children, as previously defined (secondary analyses). All analyses accounted for the complex survey design by applying the 2-y NHANES phlebotomy sample weights (WTPH2YR), cluster (SDMVPSU), and stratification (SDMVSTRA) variables when estimating SEs using Taylor series Linearization [56]. CLMD was defined as a serum magnesium concentration <2.06 mg/dL (0.85 mmol/L; 1.70 mEq/L) [2,[27], [28], [29]].

Analyses were performed using SAS software, version 9.4 (SAS Institute, Inc.). A 2-sided *P* < 0.05 was considered statistically significant. Density distributions of serum magnesium were visualized using histograms with kernel density curves, stratified by sex for children and health status groups for males and females, with the 2.5th and 97.5th percentiles indicated.

## Results

Demographic characteristics of children and adults are presented in Supplementary Tables 1 and 2. Our analyses included 787 children (*n* = 398 girls and 389 boys) and 5474 adults (*n* = 2973 females and 2501 males) (*n* = 6261). Mean serum magnesium concentrations were 1.98 (0.01) and 1.97 (0.01) mg/dL in children and adults, respectively.

**FIGURE 1:**
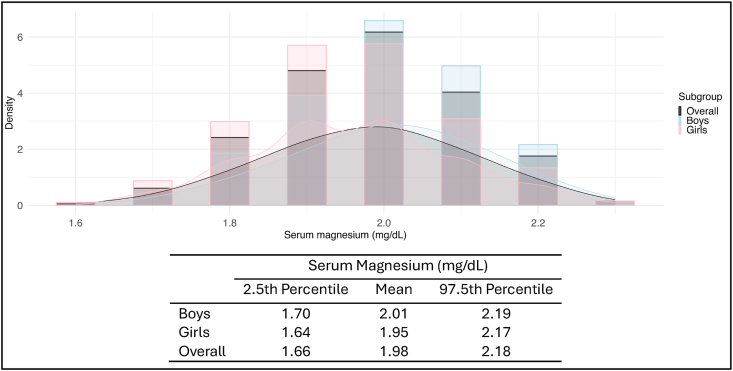
Serum magnesium concentrations (mg/dL) in children aged 12–18 y. Density distributions overlaid with kernel density curves.

Mean serum magnesium concentrations were significantly lower among girls (*P* = 0.003); however, the magnitude of this difference was nominal from a clinical standpoint (Table 1). Sensitivity analyses stratifying participants by health status yielded borderline but nonsignificant findings (*P* = 0.053). Although unhealthy children had significantly lower mean serum magnesium concentrations compared with their healthy counterparts, this result was sex-specific, with significant (clinically nonrelevant) differences in girls (*P* =0.036) but not boys (*P* =0.149) (Supplementary Table 3). These results comparing healthy and unhealthy children should be interpreted with caution, as the number of unhealthy children in the sample was small, per the CLSI guidance. In children, the serum magnesium reference interval was 1.70–2.19 mg/dL (0.70–0.90 mmol/L; 1.40–1.80 mEq/L) for boys and 1.64–2.17 mg/dL (0.68–0.90 mmol/L; 1.35–1.79 mEq/L) for girls based on data from the total population. Figure 1 illustrates the density of distributions of serum magnesium concentrations (in mg/dL) in children aged 12–18 y.

**TABLE 1:** Serum magnesium (mg/dL) reference intervals in children (aged 12–18 y)^1^

	*n*	Mean (SE)	*P*_2.5_	*P*_97.5_
Sex				
Male	398	2.01 (0.01)	1.70	2.19
Female	389	1.95 (0.01)	1.64	2.17
Combined	787	1.98 (0.01)	1.66	2.18

1 *P*_2.5_ = 2.5 percentile; *P*_97.5_ = 97.5 percentile. All children aged 12–18 y with available serum magnesium measurements were included in the analysis. Analyses were conducted using the 2-y phlebotomy weights to account for the complex survey design. Mean (SE) serum magnesium values were significantly lower in girls compared with boys (*P* = 0.003).

**FIGURE 2:**
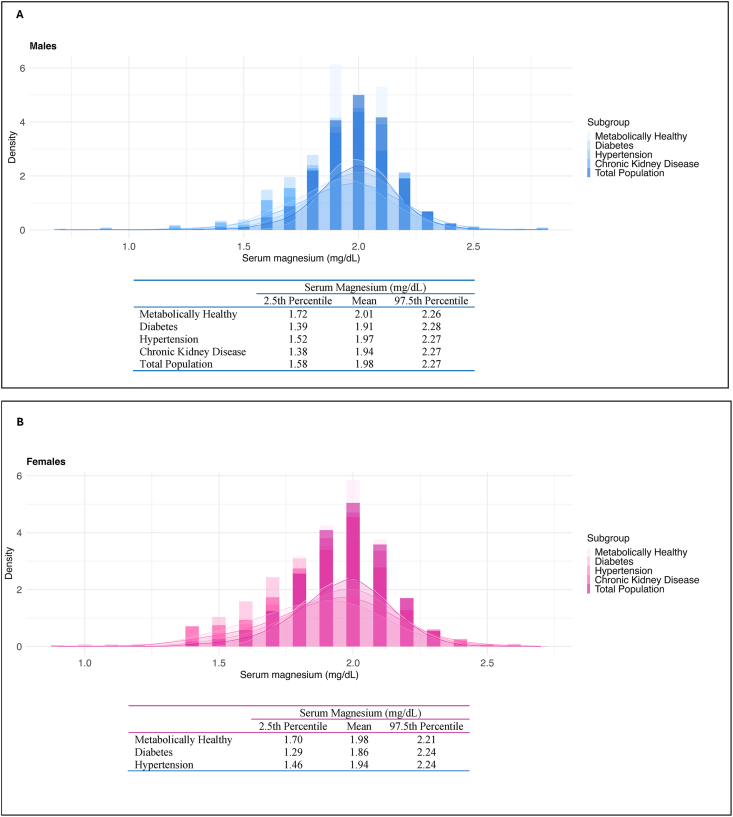
Serum magnesium concentrations (mg/dL) in males and females aged ≥19 y by health status. (A) Males. (B**)** Females. Density distributions overlaid with kernel density curves.

Mean serum magnesium concentrations were significantly lower among adults with diabetes, hypertension, or CKD compared with metabolically healthy adults (*P* <0.001, *P* = 0.003, and <0.001, respectively); however, these differences were nominal from a clinical standpoint. Mean serum magnesium concentrations for the total adult population did not differ significantly from those of metabolically healthy individuals (*P* = 0.040). Females exhibited significantly lower mean serum magnesium concentrations compared with males (*P* < 0.001), although, again, the magnitude of this difference was not clinically meaningful (Table 2). Sensitivity analyses that included participants with missing data for determining health status yielded significant results consistent with the primary findings, with significantly lower mean serum magnesium concentrations observed among adults with diabetes, hypertension, or CKD compared with metabolically healthy adults (all *P* < 0.001) (Supplementary Table 4). Serum magnesium concentrations did not significantly differ in adult males by age group (*P* = 0.251) but did in females (*P* = 0.006); however, interpretation should be made cautiously given the small sample sizes of the life-stage groups, per the CLSI guidance. After Bonferroni correction for multiple comparisons, only females aged 51–70 y had significantly higher serum magnesium concentrations compared with those aged 19–30 y (adjusted *P* = 0.015). The serum magnesium reference interval was 1.72–2.26 mg/dL (0.71–0.93 mmol/L; 1.42–1.86 mEq/L) for males and 1.70–2.21 mg/dL (0.70–0.91 mmol/L; 1.40–1.82 mEq/L) for females based on data from the metabolically healthy adult population (aged 19 y). Figure 2 shows the density of distributions of serum magnesium concentrations (in mg/dL) in adults by health status.

**TABLE 2:** Serum magnesium (mg/dL) reference intervals in adults (aged ≥19 y)^1^

Age (y)	Metabolically healthy^2^				Diabetes^3^					Hypertension^4^					Chronic kidney disease^5^					Total population				
	*N*	Mean (SE)	*P*_2.5_	*P*_97.5_	*n*	Mean (SE)	*P*_2.5_	*P*_97.5_	*P* value	*n*	Mean (SE)	*P*_2.5_	*P*_97.5_	*P* value	*n*	Mean (SE)	*P*_2.5_	*P*_97.5_	*P* value	*n*	Mean (SE)	*P*_2.5_	*P*_97.5_	*P* value
Males																								
≥19	173	2.01 (0.01)	1.72	2.26	510	1.91 (0.01)	1.39	2.28	<0.001	1293	1.97 (0.01)	1.52	2.27	0.012	489	1.94 (0.02)	1.38	2.27	0.007	2501	1.98 (0.01)	1.58	2.27	0.071
19–30	59	1.97 (0.02)	1.70	2.18	8	1.85 (0.07)	1.60	2.16	0.183	52	1.97 (0.02)	1.61	2.32	0.844	9	1.88 (0.06)	1.70	2.00	0.275	324	1.97 (0.01)	1.63	2.27	0.940
31–50	77	2.03 (0.02)	1.80	2.28	60	1.99 (0.03)	1.52	2.44	0.229	211	1.98 (0.02)	1.58	2.29	0.062	31	1.95 (0.05)	1.60	2.31	0.192	659	2.00 (0.01)	1.63	2.27	0.137
51–70	32	2.02 (0.04)	1.80	2.28	277	1.89 (0.02)	1.35	2.23	0.003	666	1.96 (0.01)	1.44	2.25	0.155	212	1.93 (0.02)	1.17	2.24	0.039	1010	1.98 (0.01)	1.53	2.25	0.270
≥71	5	2.15 (0.06)	2.00	2.29	165	1.90 (0.02)	1.34	2.26	0.002	364	1.97 (0.01)	1.51	2.29	0.010	237	1.96 (0.02)	1.38	2.30	0.005	508	1.98 (0.01)	1.50	2.30	0.016
Females																								
≥19	197	1.98 (0.01)	1.70	2.21	517	1.86 (0.01)	1.29	2.24	<0.001	1340	1.94 (0.01)	1.46	2.24	0.029	526	1.91 (0.01)	1.33	2.28	0.002	2973	1.96 (0.01)	1.53	2.23	0.185
19–30	68	1.95 (0.02)	1.70	2.10	11	1.86 (0.05)	1.50	2.06	0.138	29	1.93 (0.03)	1.61	2.16	0.552	19	1.95 (0.04)	1.60	2.09	0.982	376	1.94 (0.01)	1.61	2.16	0.496
31–50	79	1.99 (0.02)	1.70	2.20	90	1.86 (0.02)	1.42	2.18	<0.001	204	1.93 (0.02)	1.51	2.19	0.045	72	1.89 (0.02)	1.46	2.35	0.004	839	1.96 (0.01)	1.60	2.20	0.117
51–70	48	2.00 (0.03)	1.70	2.25	271	1.86 (0.02)	1.29	2.19	0.002	710	1.96 (0.01)	1.47	2.25	0.177	220	1.92 (0.02)	1.41	2.28	0.040	1233	1.98 (0.01)	1.51	2.26	0.428
≥71	2	2.19 (0.11)	1.90	2.29	145	1.85 (0.03)	1.24	2.29	0.010	397	1.93 (0.01)	1.38	2.26	0.036	215	1.90 (0.02)	1.24	2.27	0.022	525	1.95 (0.01)	1.36	2.27	0.045

Mean (SE) serum magnesium values were significantly lower in adult (≥19 y) females compared with males (*P* < 0.001).

1 *P*_2.5_ = 2.5 percentile, *P*_97.5_ = 97.5 percentile. Participants with missing values for determining health status were excluded from the analyses. All analyses were conducted using the 2-y phlebotomy weights to account for the complex survey design.

2 Metabolically healthy adults were defined as participants without type 2 diabetes, hypertension, or chronic kidney disease who met all of the following criteria: *1*) systolic blood pressure <120 mm Hg and diastolic blood pressure <80 mm Hg; *2*) fasting plasma glucose <100 mg/dL (<5.6 mmol/L) and hemoglobin A1c <5.7%; 3) triglycerides <150 mg/dL, HDL-C ≥40 mg/dL in males and ≥50 mg/dL in females, and waist circumference <102 cm for males and <88 cm for females (Asian-specific cutoffs: <90 cm for males and <80 cm for females); and *4*) no self-reported use of medications for high cholesterol, hypertension, or diabetes.

3 Diabetes was defined by self-reported physician-diagnosed type 2 diabetes, self-reported use of antidiabetic medication, fasting plasma glucose ≥126 mg/dL (≥7.0 mmol/L), or hemoglobin A1c ≥6.5%.

4 Hypertension was defined by self-reported physician-diagnosed hypertension, use of antihypertensive medication, or systolic blood pressure ≥130 mm Hg or diastolic blood pressure ≥85 mm Hg.

5 Chronic kidney disease was defined by estimated glomerular filtration rate <60 mL/min/1.73 m^2^ or an albumin-to-creatinine ratio >30 mg/g.

On the basis of these nationally representative data, we estimate that the survey-weighed proportion of adults in the United States who are at risk of CLMD to be 67.8% (65.8% and 69.8% in males and females, respectively), as indicated by a serum magnesium concentration of <2.07 mg/dL (0.85 mmol/L; 1.70 mEq/L) [27,29]. The survey-weighed prevalence of CLMD in metabolically healthy adults was 66.6% (65.8% in males and 69.8% in females), compared with 68.5% (66.9% in males and 70.2% in females) in those with hypertension, 78.3% (75.9% in males and 80.9% in females) in those with diabetes, and 71.1% (69.6% in males and 72.4% in females) in those with CKD.

## Discussion

Assessment of patient magnesium status is increasingly recognized as an important component of clinical evaluation and monitoring in the healthcare setting. Although imperfect, serum magnesium is a readily available, easily measured, and practical clinical biomarker alternative to the magnesium load (tolerance/retention) test that could be incorporated into a standard metabolic panel. Our findings reflect current sex-specific nationally representative data and contemporary statistical approaches recommended by the IFCC [53]. These findings support a more broad population-based reference interval than the former interval of 1.82–2.32 mg/dL (0.75–0.96 mmol/L; 1.50–1.91 mEq/L) for adults proposed by Lowenstein and Stanton [41] in 1986 using data from NHANES I (1971–1974), granted the former was based on the 2.5th and 97.5th percentiles and the latter on the 5th and 95th percentiles. In addition, our analyses suggest that a substantial portion of the US population may have or be at risk of CLMD, indicated by a serum magnesium concentration <2.06 mg/dL (0.85 mmol/L; 1.70 mEq/L), which has been recognized as important for preventing long-term subclinical ailments that can lead or contribute to the commencement and progression of many chronic diseases. The noticeably higher proportion of adults with diabetes and a serum magnesium concentration indicative of CLMD in this study supports longstanding consensus of this biomarker as a pragmatic screening tool to prompt closer evaluation and monitoring of magnesium status in this patient subpopulation. Magnesium is known to play a central role in glucose metabolism and insulin signaling, which provides biological plausibility to these observed findings in adults with diabetes.

The subtle decrease in mean serum magnesium concentrations in the population since NHANES I (1971–1974) is not surprising and likely reflects a multitude of factors. The magnesium content of fruits and vegetables has decreased over the last 50 y, after the content in soil used for farming, and ∼80% of this mineral is lost during food processing [57]. Lower mean serum magnesium concentrations and the relatively high prevalence of adults with or at risk of CLMD also likely reflect suboptimal dietary magnesium intake, relative to national recommendations, by about half of the US population [1,31,32]. However, these effects may be somewhat offset on a population level by the increased use of magnesium-containing dietary supplements and over-the-counter (OTC) antacid medications, as well as the higher consumption of fortified and enriched foods relative to >50 y ago. The current higher prevalence of metabolic diseases and concomitant increased use of prescription and OTC medications that promote magnesium wasting may also predispose more individuals to a lower serum status [6]. It is likely that other changes, such as, but not limited to, shifts in population demographics and improvements in the healthcare system in the United States and its utilization, also affect differences in serum magnesium status of the population over the last 5 decades. However, it is important to note that several methodological and contextual differences between the values presented here and those by Lowenstein and Stanton [41] limit direct comparability of results. First, population-based reference intervals derived from NHANES I were based on the 5th and 95th percentiles of the total population (sexes combined), whereas the reference intervals presented in this work represent sex-specific 2.5th and 97.5th percentiles, per current recommendations from the IFCC, of metabolically healthy adults. Moreover, serum magnesium concentrations in NHANES I [41] were quantified using atomic absorption spectroscopy, whereas a photometric colorimetric assay based on the xylidyl blue reaction was used to quantify levels in the 2021–2023 NHANES data cycles [51,52]. Although these methods generally yield comparable results, the risk of increased interlaboratory variation is present.

Our study has several notable strengths. We agreed to and did not deviate from a statistical analysis plan before downloading the raw data, which enhances transparency of findings and limits the influence of investigator bias. The use of a large and nationally representative dataset enhances the generalizability of our findings. Application of contemporary statistical approaches recommended by the IFCC in metabolically healthy adults likely increases the clinical relevance of the reference intervals. Sensitivity analyses confirmed the robustness of our primary findings. Our analyses also highlight the importance of serum magnesium as a pragmatic screener to prompt closer evaluation and monitoring of magnesium status when CLMD risk is indicated, particularly within adult subpopulations with diabetes.

Several limitations in this work also exist. Although the NHANES is large and nationally representative, sample size constraints limited our confidence within certain sex-specific life-stage categories where serum magnesium concentrations were available for <120 individuals. Sample size limitations impeded stratification by race and ethnicity. Future studies with larger sample sizes will be necessary to explore demographic-specific reference intervals and further refine risk thresholds. In forthcoming work, we will examine the relationship of circulating serum magnesium concentrations with estimated dietary magnesium intake, the newly developed magnesium depletion score, and health biomarkers.

In conclusion, measurement of serum magnesium is an important component of clinical evaluation and monitoring in the healthcare setting. This study provides more narrow and current population-based reference intervals for serum magnesium, and these data highlight the potential widespread prevalence of CLMD across the US population. Future larger cohorts are needed to fully explore demographic-specific reference intervals and variables that may affect serum magnesium concentrations in subpopulations. Controlled metabolic studies that collect data from a magnesium load (tolerance/retention) test and assess serum magnesium can provide better insight into the reliability of both serum magnesium concentrations for assessment of magnesium status and use of the widely accepted CLMD threshold for preventing or treating metabolic disease.

## Author contributions

The authors’ responsibilities were as follows – RC, JG, AR, TCW: designed research; KJ, TCW: conducted research; TCW: provided essential materials; KJ: analyzed data or performed statistical analysis; KJ, TCW: wrote the paper; TCW: had primary responsibility for the final content; and all authors: read and approved the final manuscript.

## Declaration of generative AI statement

The authors declare that no generative AI or AI-assisted technologies were used in the writing of this manuscript.

## Data availability

Data described in the manuscript, code book, and analytic code will be made available upon reasonable request.

## Funding

Funding for this study was provided through an unrestricted educational grant from Pharmavite, LLC to Think Healthy Group, LLC. This funding was solely used to support tuition for the student conducting the analyses, and no salaries or compensation were taken by the authors or their respective institutions. The funder had no role in the design of the study; in the collection, analyses, or interpretation of data; or in the writing of the manuscript.

## Conflict of interest

RC is a Senior Research Fellow of the Center for Magnesium Education and Research (CMER). AR is the Director of the CMER. TCW has received competitive unrestricted research grants unrelated to this work from New Capstone, Inc. TCW receives royalties from the Academy of Nutrition and Dietetics for editing *the Health Professionals Guide to Dietary Supplements*. TCW is on the science advisory boards of Forbes Health (unpaid), Haleon, and Deerland Probiotics & Enzymes, and is a member of the National Academy of Medicine Expert Panel on Validating Health Information Using GenAI: A Nutrition Case Study (unpaid). TCW is the Editor-in-Chief of the *Journal of Dietary Supplements*, Deputy Editor-in-Chief of the *Journal of the American Nutrition Association*, and Nutrition Section Editor of *Annals of Medicine*. TCW is a Senior Fellow of the CMER (unpaid). TCW has an up-to-date International Committee of Medical Journal Editors disclosure of interest statement available on his website (http://www.drtaylorwallace.com). The other authors report no conflicts of interest.
