# Supplementary material for: Serum Magnesium Concentrations in the United States—An Updated Population Reference Interval in Children and Adults
Source: J Nutr. 2026 Apr 16;156(6):101539. doi: 10.1016/j.tjnut.2026.101539 (PMC13279296; doi:10.1016/j.tjnut.2026.101539)
Supplement: Multimedia component 1 [file mmc1.docx]

Serum magnesium levels in the United States—an updated population reference interval in children and adults

Keyi Jiao, Rebecca Costello, Jaime Gahche, Andrea Rosanoff, and Taylor C. Wallace

**Supplemental Table 1:** Baseline characteristics of children 12-18 y.

|  | ***n*** | **%** | **Mean (SE)** |
| --- | --- | --- | --- |
| Sex |  |  |  |
| Male | 398 | 51.8 | -- |
| Female | 389 | 48.2 | -- |
| Age, y | -- | -- | 15.0 (0.07) |
| Race and Ethnicity |  |  |  |
| Asian | 46 | 8.3 | -- |
| Black | 114 | 12.5 | -- |
| Hispanic | 261 | 25.0 | -- |
| White | 296 | 45.0 | -- |
| Mixed and Other | 70 | 9.1 | -- |
| Serum magnesium, mg/dL | -- | -- | 1.98 (0.01) |

**Supplemental Table 2:** Baseline characteristics of adults (aged ≥19 y).

|  | ***n*** | **%** | **Mean (SE)** |
| --- | --- | --- | --- |
| Sex |  |  |  |
| Male | 2,501 | 49.4 | -- |
| Female | 2,973 | 50.6 | -- |
| Age | -- | -- | 48.7 (0.24) |
| Age Groups |  |  |  |
| 19-30 y | 700 | 19.2 | -- |
| 31-50 y | 1,498 | 33.6 | -- |
| 51-70 y | 2,243 | 33.9 | -- |
| 71+ y | 1,033 | 13.3 | -- |
| Race and Ethnicity |  |  |  |
| Asian | 302 | 6.2 | -- |
| Black | 632 | 10.7 | -- |
| Hispanic | 932 | 16.7 | -- |
| White | 3,270 | 60.6 | -- |
| Mixed and Other | 338 | 5.8 | -- |
| Serum magnesium, mg/dL | -- | -- | 1.97 (0.01) |

**Supplementary Table 3.** Sensitivity analysis of serum magnesium reference intervals in children (age 12–18 y) by health status.^1^

| **Group** | **Sex** | ***n*^2^** | **Mean (SE)** | **P_2.5_** | **P_97.5_** |
| --- | --- | --- | --- | --- | --- |
| Healthy^3^ | Boys | 348 | 2.01 (0.01) | 1.70 | 2.19 |
|  | Girls | 342 | 1.96 (0.01) | 1.70 | 2.17 |
|  | Combined | 690 | 1.99 (0.01) | 1.70 | 2.18 |
| Unhealthy | Boys | 50 | 1.98 (0.02) | 1.70 | 2.13 |
|  | Girls | 47 | 1.90 (0.02) | 1.60 | 2.08 |
|  | Combined | 97 | 1.94 (0.01) | 1.61 | 2.10 |

^1^P_2.5_ = 2.5 percentile; P_97.5_ = 97.5 percentile. All analyses were conducted using the 2-y phlebotomy weights to account for the complex survey design.
^2^Unweighted sample size.
^3^Children were considered generally healthy if they met all of the following criteria NHANES medical condition questionnaires: no history of asthma and no evidence of liver-related conditions (including viral hepatitis [A, B, or C]), autoimmune liver diseases (e.g., primary biliary cirrhosis, autoimmune hepatitis, or sclerosing cholangitis), genetic liver disorders (e.g., alpha-1-antitrypsin deficiency, hemochromatosis, or Wilson’s disease), drug- or medication-induced liver injury, alcoholic liver disease, nonalcoholic fatty liver disease or fatty liver disease, liver cancer, liver cysts or abscess, liver fibrosis, or liver cirrhosis. Children with missing values for determining health status were included in the healthy group.

**Supplementary Table 4.** Sensitivity analysis of serum magnesium reference intervals in adults (≥19 y).^1^

| **Age, y** | **Metabolically healthy^2^** | | | | **Diabetes^3^** | | | | | **Hypertension^4^** | | | | | **Chronic kidney disease^5^** | | | | | **Total population** | | | | |
| --- | --- | --- | --- | --- | --- | --- | --- | --- | --- | --- | --- | --- | --- | --- | --- | --- | --- | --- | --- | --- | --- | --- | --- | --- |
|  | ***n*** | **Mean (SE)** | **P_2.5_** | **P_97.5_** | ***n*** | **Mean (SE)** | **P_2.5_** | **P_97.5_** | ***P* value** | ***n*** | **Mean (SE)** | **P_2.5_** | **P_97.5_** | ***P* value** | ***n*** | **Mean (SE)** | **P_2.5_** | **P_97.5_** | ***P* value** | ***n*** | **Mean (SE)** | **P_2.5_** | **P_97.5_** | ***P* value** |
| **Men** |  |  |  |  |  |  |  |  |  |  |  |  |  |  |  |  |  |  |  |  |  |  |  |  |
| ≥19 | 404 | 2.00 (0.01) | 1.69 | 2.25 | 510 | 1.90 (0.02) | 1.38 | 2.27 | <0.001 | 1,293 | 1.97 (0.01) | 1.51 | 2.29 | 0.011 | 489 | 1.93 (0.02) | 1.14 | 2.25 | 0.009 | 2,501 | 1.98 (0.01) | 1.58 | 2.27 | 0.100 |
| 19–30 | 143 | 1.97 (0.01) | 1.63 | 2.19 | 8 | 1.85 (0.08) | 1.60 | 2.13 | 0.131 | 52 | 2.00 (0.02) | 1.80 | 2.33 | 0.810 | 9 | 1.89 (0.07) | 1.70 | 1.99 | 0.234 | 324 | 1.97 (0.01) | 1.63 | 2.27 | 0.926 |
| 31–50 | 162 | 2.02 (0.01) | 1.71 | 2.26 | 60 | 2.02 (0.04) | 1.70 | 2.43 | 0.333 | 211 | 2.01 (0.03) | 1.70 | 2.42 | 0.028 | 31 | 1.96 (0.03) | 1.70 | 2.09 | 0.207 | 659 | 2.00 (0.01) | 1.63 | 2.27 | 0.060 |
| 51–70 | 81 | 2.02 (0.02) | 1.80 | 2.27 | 277 | 1.88 (0.03) | 1.24 | 2.24 | <0.001 | 666 | 1.94 (0.02) | 1.29 | 2.27 | 0.021 | 212 | 1.90 (0.04) | 0.82 | 2.26 | 0.004 | 1,010 | 1.98 (0.01) | 1.53 | 2.25 | 0.065 |
| ≥71 | 18 | 2.10 (0.04) | 1.90 | 2.28 | 165 | 1.90 (0.03) | 1.34 | 2.21 | <0.001 | 364 | 1.98 (0.01) | 1.53 | 2.26 | 0.008 | 237 | 1.97 (0.02) | 1.35 | 2.27 | 0.005 | 508 | 1.98 (0.01) | 1.50 | 2.30 | 0.016 |
| **Women** |  |  |  |  |  |  |  |  |  |  |  |  |  |  |  |  |  |  |  |  |  |  |  |  |
| ≥19 | 418 | 1.99 (0.01) | 1.65 | 2.22 | 517 | 1.87 (0.01) | 1.30 | 2.24 | <0.001 | 1,340 | 1.95 (0.01) | 1.48 | 2.27 | <0.001 | 526 | 1.93 (0.01) | 1.41 | 2.31 | <0.001 | 2,973 | 1.96 (0.01) | 1.53 | 2.23 | <0.001 |
| 19–30 | 132 | 1.96 (0.01) | 1.63 | 2.17 | 11 | 1.82 (0.06) | 1.60 | 1.90 | 0.082 | 29 | 1.91 (0.05) | 1.70 | 2.24 | 0.448 | 19 | 1.95 (0.04) | 1.70 | 2.09 | 0.904 | 376 | 1.94 (0.01) | 1.61 | 2.16 | 0.076 |
| 31–50 | 173 | 2.01 (0.01) | 1.70 | 2.24 | 90 | 1.89 (0.03) | 1.46 | 2.10 | <0.001 | 204 | 1.96 (0.02) | 1.58 | 2.24 | <0.001 | 72 | 1.93 (0.04) | 1.43 | 2.32 | <0.001 | 839 | 1.96 (0.01) | 1.60 | 2.20 | <0.001 |
| 51–70 | 100 | 2.02 (0.02) | 1.69 | 2.28 | 271 | 1.87 (0.02) | 1.21 | 2.20 | <0.001 | 710 | 1.96 (0.01) | 1.47 | 2.27 | 0.011 | 220 | 1.94 (0.04) | 1.40 | 2.35 | 0.002 | 1,233 | 1.98 (0.01) | 1.51 | 2.26 | 0.052 |
| ≥71 | 13 | 2.08 (0.05) | 1.80 | 2.28 | 145 | 1.85 (0.03) | 1.28 | 2.32 | <0.001 | 397 | 1.94 (0.02) | 1.44 | 2.28 | 0.006 | 215 | 1.92 (0.02) | 1.41 | 2.28 | 0.003 | 525 | 1.95 (0.01) | 1.36 | 2.27 | 0.011 |

^1^P_2.5_ = 2.5 percentile; P_97.5_ = 97.5 percentile. All analyses were conducted using the 2-y phlebotomy weights to account for the complex survey design.

^2^Metabolically healthy adults were defined as participants without type 2 diabetes, hypertension, or chronic kidney disease who met all of the following criteria: systolic blood pressure <130 mm Hg and diastolic blood pressure <85 mm Hg; fasting plasma glucose <100 mg/dL (<5.6 mmol/L); triglycerides <150 mg/dL; HDL cholesterol ≥40 mg/dL in males and ≥50 mg/dL in females; no self-reported use of medications for high cholesterol, hypertension, or diabetes; and waist circumference <102 cm for men and <88 cm for women (Asian-specific cutoffs: <90 cm for men and <80 cm for women). Participants with missing values for determining health status were included in the metabolically healthy group if they met at least three of the criteria.

^3^Diabetes was defined by self-reported physician-diagnosed type 2 diabetes, self-reported use of antidiabetic medication, fasting plasma glucose ≥7.0 mmol/L (≥126 mg/dL), or hemoglobin A1c ≥6.5%.

^4^Hypertension was defined by self-reported physician-diagnosed hypertension, use of antihypertensive medication, or systolic blood pressure ≥130 mm Hg or diastolic blood pressure ≥85 mm Hg.

^5^Chronic kidney disease was defined by estimated glomerular filtration rate <60 mL/min/1.73 m² or an albumin-to-creatinine ratio >30 mg/g.

**
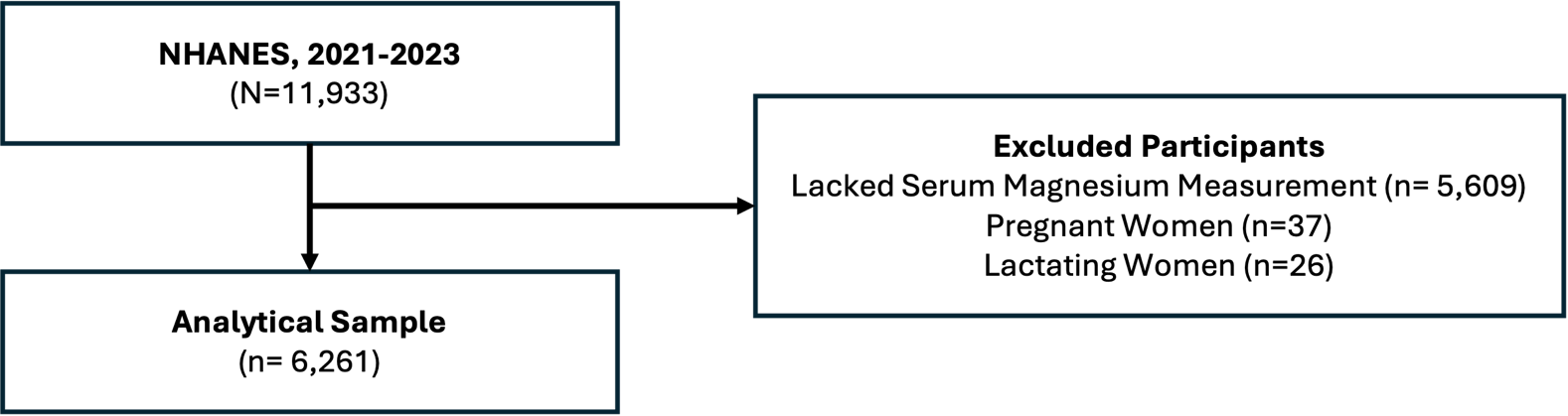
**

**Supplementary Figure 1:** Participant flow diagram.

**Supplementary Material:** Prespecified Statistical Analysis Plan

Approved by authors RC, JG, AR, and TCW on **11 September 2025.**

**Objective:**

Determine the reference range for serum magnesium among children (age 12-18 years) and adults (age 19+ years) enrolled in the 2021-2023 data cycles of NHANES:

*Children (Shell Table 1):*

- Total population (*reference category*)

*Adults (Shell Table 3):*

- Metabolically healthy adults^1^ (reference category)
- Adults with diabetes^2^
- Adults with hypertension^3^
- Adults with chronic kidney disease^4^
- Total population

^1^Metabolically healthy adults were defined as participants without type 2 diabetes, hypertension, or chronic kidney disease who met all of the following criteria: 1) systolic blood pressure <120 mm Hg and diastolic blood pressure <80 mm Hg; 2) fasting plasma glucose <100 mg/dL (<5.6 mmol/L) and hemoglobin A1c <5.7%; 3) triglycerides <150 mg/dL, HDL-C ≥40 mg/dL in males and ≥50 mg/dL in females, and waist circumference <102 cm for males and <88 cm for females (Asian-specific cutoffs: <90 cm for males and <80 cm for females); and 4) no self-reported use of medications for high cholesterol, hypertension, or diabetes.

^2^Diabetes was defined by self-reported physician-diagnosed type 2 diabetes, self-reported use of antidiabetic medication, fasting plasma glucose ≥126 mg/dL (≥7.0 mmol/L), or HbA1c ≥6.5%.

^3^Hypertension was defined by self-reported physician-diagnosed hypertension, use of antihypertensive medication, or systolic blood pressure ≥130 mm Hg or diastolic blood pressure ≥85 mm Hg.

^4^Chronic kidney disease was defined by estimated glomerular filtration rate <60 mL/min/1.73 m² or an albumin-to-creatinine ratio >30 mg/g.

**Exclusions:**

- Lacking serum magnesium measurement (LBXMAGN)
- Pregnant women (RHD143 and RIDEXPRG)
- Lactating women (RHQ200)

**Sensitivity Analyses:**

*Children (Shell Table 2):*

- Healthy^*^
- Unhealthy

^*^Children were considered generally healthy if they met all of the following criteria NHANES medical condition questionnaires: no history of asthma and no evidence of liver-related conditions (including viral hepatitis [A, B, or C]), autoimmune liver diseases (e.g., primary biliary cirrhosis, autoimmune hepatitis, or sclerosing cholangitis), genetic liver disorders (e.g., alpha-1-antitrypsin deficiency, hemochromatosis, or Wilson’s disease), drug- or medication-induced liver injury, alcoholic liver disease, nonalcoholic fatty liver disease or fatty liver disease, liver cancer, liver cysts or abscess, liver fibrosis, or liver cirrhosis. Children with missing values for determining health status were included in the healthy group.

*Adults (Shell Table 4):*

- Metabolically healthy adults^*1^ (reference category)
- Adults with diabetes^2^
- Adults with hypertension^3^
- Adults with chronic kidney disease^4^
- Total population

^*^Participants with missing values for determining health status were included in the metabolically healthy group if they met at least three of the criteria.

^1^Metabolically healthy adults were defined as participants without type 2 diabetes, hypertension, or chronic kidney disease who met all of the following criteria: 1) systolic blood pressure <120 mm Hg and diastolic blood pressure <80 mm Hg; 2) fasting plasma glucose <100 mg/dL (<5.6 mmol/L) and hemoglobin A1c <5.7%; 3) triglycerides <150 mg/dL, HDL-C ≥40 mg/dL in males and ≥50 mg/dL in females, and waist circumference <102 cm for males and <88 cm for females (Asian-specific cutoffs: <90 cm for males and <80 cm for females); and 4) no self-reported use of medications for high cholesterol, hypertension, or diabetes.

^2^Diabetes was defined by self-reported physician-diagnosed type 2 diabetes, self-reported use of antidiabetic medication, fasting plasma glucose ≥126 mg/dL (≥7.0 mmol/L), or HbA1c ≥6.5%.

^3^Hypertension was defined by self-reported physician-diagnosed hypertension, use of antihypertensive medication, or systolic blood pressure ≥130 mm Hg or diastolic blood pressure ≥85 mm Hg.

^4^Chronic kidney disease was defined by estimated glomerular filtration rate <60 mL/min/1.73 m² or an albumin-to-creatinine ratio >30 mg/g.

**Shell Table 1.** Serum magnesium reference intervals in children (aged 12–18 y).^1^

|  | ***n*** | **Mean (SE)** | **P_2.5_** | **P_97.5_** |
| --- | --- | --- | --- | --- |
| Sex |  |  |  |  |
| Male |  |  |  |  |
| Female |  |  |  |  |
| Combined |  |  |  |  |

^1^P_2.5_ = 2.5 percentile; P_97.5_ = 97.5 percentile. All children aged 12–18 y with available serum magnesium measurements will be included in the analysis. Analyses will be conducted using the 2-y phlebotomy weights to account for the complex survey design.

**Shell Table 2.** Sensitivity analysis of serum magnesium reference intervals in children (age 12–18 y) by health status.^1^

| **Group** | **Sex** | ***n*^2^** | **Mean (SE)** | **P_2.5_** | **P_97.5_** |
| --- | --- | --- | --- | --- | --- |
| Healthy^3^ | Boys | 348 | 2.01 (0.01) | 1.70 | 2.19 |
|  | Girls | 342 | 1.96 (0.01) | 1.70 | 2.17 |
|  | Combined | 690 | 1.99 (0.01) | 1.70 | 2.18 |
| Unhealthy | Boys | 50 | 1.98 (0.02) | 1.70 | 2.13 |
|  | Girls | 47 | 1.90 (0.02) | 1.60 | 2.08 |
|  | Combined | 97 | 1.94 (0.01) | 1.61 | 2.10 |

^1^P_2.5_ = 2.5 percentile; P_97.5_ = 97.5 percentile. All analyses will be conducted using the 2-y phlebotomy weights to account for the complex survey design.
^2^Unweighted sample size.
^3^Children will be considered generally healthy if they met all of the following criteria NHANES medical condition questionnaires: no history of asthma and no evidence of liver-related conditions (including viral hepatitis [A, B, or C]), autoimmune liver diseases (e.g., primary biliary cirrhosis, autoimmune hepatitis, or sclerosing cholangitis), genetic liver disorders (e.g., alpha-1-antitrypsin deficiency, hemochromatosis, or Wilson’s disease), drug- or medication-induced liver injury, alcoholic liver disease, nonalcoholic fatty liver disease or fatty liver disease, liver cancer, liver cysts or abscess, liver fibrosis, or liver cirrhosis. Children with missing values for determining health status will be included in the healthy group.

**Shell Table 3.** Serum magnesium reference intervals in adults (aged ≥19 y).^1^

| **Age, y** | **Metabolically healthy**^2^ | | | | **Diabetes**^3^ | | | | | **Hypertension**^4^ | | | | | **Chronic kidney disease**^5^ | | | | | **Total population** | | | | |
| --- | --- | --- | --- | --- | --- | --- | --- | --- | --- | --- | --- | --- | --- | --- | --- | --- | --- | --- | --- | --- | --- | --- | --- | --- |
|  | ***n*** | **Mean (SE)** | **P_2.5_** | **P_97.5_** | ***n*** | **Mean (SE)** | **P_2.5_** | **P_97.5_** | ***P* value** | ***n*** | **Mean (SE)** | **P_2.5_** | **P_97.5_** | ***P* value** | ***n*** | **Mean (SE)** | **P_2.5_** | **P_97.5_** | ***P* value** | ***n*** | **Mean (SE)** | **P_2.5_** | **P_97.5_** | ***P* value** |
| **Men** |  |  |  |  |  |  |  |  |  |  |  |  |  |  |  |  |  |  |  |  |  |  |  |  |
| >19 |  |  |  |  |  |  |  |  |  |  |  |  |  |  |  |  |  |  |  |  |  |  |  |  |
| 19–30 |  |  |  |  |  |  |  |  |  |  |  |  |  |  |  |  |  |  |  |  |  |  |  |  |
| 31–50 |  |  |  |  |  |  |  |  |  |  |  |  |  |  |  |  |  |  |  |  |  |  |  |  |
| 51–70 |  |  |  |  |  |  |  |  |  |  |  |  |  |  |  |  |  |  |  |  |  |  |  |  |
| ≥71 |  |  |  |  |  |  |  |  |  |  |  |  |  |  |  |  |  |  |  |  |  |  |  |  |
| **Women** |  |  |  |  |  |  |  |  |  |  |  |  |  |  |  |  |  |  |  |  |  |  |  |  |
| >19 |  |  |  |  |  |  |  |  |  |  |  |  |  |  |  |  |  |  |  |  |  |  |  |  |
| 19–30 |  |  |  |  |  |  |  |  |  |  |  |  |  |  |  |  |  |  |  |  |  |  |  |  |
| 31–50 |  |  |  |  |  |  |  |  |  |  |  |  |  |  |  |  |  |  |  |  |  |  |  |  |
| 51–70 |  |  |  |  |  |  |  |  |  |  |  |  |  |  |  |  |  |  |  |  |  |  |  |  |
| ≥71 |  |  |  |  |  |  |  |  |  |  |  |  |  |  |  |  |  |  |  |  |  |  |  |  |

^1^P_2.5_ = 2.5 percentile, P_97.5_ = 97.5 percentile. Participants with missing values for determining health status will be excluded from the analyses. All analyses will be conducted using the 2-year phlebotomy weights to account for the complex survey design.

^2^Metabolically healthy adults were defined as participants without type 2 diabetes, hypertension, or chronic kidney disease who met all of the following criteria: 1) systolic blood pressure <120 mm Hg and diastolic blood pressure <80 mm Hg; 2) fasting plasma glucose <100 mg/dL (<5.6 mmol/L) and hemoglobin A1c <5.7%; 3) triglycerides <150 mg/dL, HDL-C ≥40 mg/dL in males and ≥50 mg/dL in females, and waist circumference <102 cm for males and <88 cm for females (Asian-specific cutoffs: <90 cm for males and <80 cm for females); and 4) no self-reported use of medications for high cholesterol, hypertension, or diabetes.

^3^Diabetes will be defined by self-reported physician-diagnosed type 2 diabetes, self-reported use of antidiabetic medication, fasting plasma glucose ≥126 mg/dL (≥7.0 mmol/L), or HbA1c ≥6.5%.

^4^Hypertension will be defined by self-reported physician-diagnosed hypertension, use of antihypertensive medication, or systolic blood pressure ≥130 mm Hg or diastolic blood pressure ≥85 mm Hg.

^5^Chronic kidney disease will be defined by estimated glomerular filtration rate <60 mL/min/1.73 m² or an albumin-to-creatinine ratio >30 mg/g.

**Shell Table 4.** Sensitivity analysis of serum magnesium reference intervals in adults (≥19 y).^1^

| **Age, y** | **Metabolically healthy^2^** | | | | **Diabetes^3^** | | | | | **Hypertension^4^** | | | | | **Chronic kidney disease^5^** | | | | | **Total population** | | | | |
| --- | --- | --- | --- | --- | --- | --- | --- | --- | --- | --- | --- | --- | --- | --- | --- | --- | --- | --- | --- | --- | --- | --- | --- | --- |
|  | ***n*** | **Mean (SE)** | **P_2.5_** | **P_97.5_** | ***n*** | **Mean (SE)** | **P_2.5_** | **P_97.5_** | ***P* value** | ***n*** | **Mean (SE)** | **P_2.5_** | **P_97.5_** | ***P* value** | ***n*** | **Mean (SE)** | **P_2.5_** | **P_97.5_** | ***P* value** | ***n*** | **Mean (SE)** | **P_2.5_** | **P_97.5_** | ***P* value** |
| **Men** |  |  |  |  |  |  |  |  |  |  |  |  |  |  |  |  |  |  |  |  |  |  |  |  |
| ≥19 |  |  |  |  |  |  |  |  |  |  |  |  |  |  |  |  |  |  |  |  |  |  |  |  |
| 19–30 |  |  |  |  |  |  |  |  |  |  |  |  |  |  |  |  |  |  |  |  |  |  |  |  |
| 31–50 |  |  |  |  |  |  |  |  |  |  |  |  |  |  |  |  |  |  |  |  |  |  |  |  |
| 51–70 |  |  |  |  |  |  |  |  |  |  |  |  |  |  |  |  |  |  |  |  |  |  |  |  |
| ≥71 |  |  |  |  |  |  |  |  |  |  |  |  |  |  |  |  |  |  |  |  |  |  |  |  |
| **Women** |  |  |  |  |  |  |  |  |  |  |  |  |  |  |  |  |  |  |  |  |  |  |  |  |
| ≥19 |  |  |  |  |  |  |  |  |  |  |  |  |  |  |  |  |  |  |  |  |  |  |  |  |
| 19–30 |  |  |  |  |  |  |  |  |  |  |  |  |  |  |  |  |  |  |  |  |  |  |  |  |
| 31–50 |  |  |  |  |  |  |  |  |  |  |  |  |  |  |  |  |  |  |  |  |  |  |  |  |
| 51–70 |  |  |  |  |  |  |  |  |  |  |  |  |  |  |  |  |  |  |  |  |  |  |  |  |
| ≥71 |  |  |  |  |  |  |  |  |  |  |  |  |  |  |  |  |  |  |  |  |  |  |  |  |

^1^P_2.5_ = 2.5 percentile; P_97.5_ = 97.5 percentile. All analyses will be conducted using the 2-y phlebotomy weights to account for the complex survey design.

^2^Metabolically healthy adults will be defined as participants without type 2 diabetes, hypertension, or chronic kidney disease who met all of the following criteria: systolic blood pressure <130 mm Hg and diastolic blood pressure <85 mm Hg; fasting plasma glucose <100 mg/dL (<5.6 mmol/L); triglycerides <150 mg/dL; HDL cholesterol ≥40 mg/dL in males and ≥50 mg/dL in females; no self-reported use of medications for high cholesterol, hypertension, or diabetes; and waist circumference <102 cm for men and <88 cm for women (Asian-specific cutoffs: <90 cm for men and <80 cm for women). Participants with missing values for determining health status will be included in the metabolically healthy group if they met at least three of the criteria.

^3^Diabetes will be defined by self-reported physician-diagnosed type 2 diabetes, self-reported use of antidiabetic medication, fasting plasma glucose ≥7.0 mmol/L (≥126 mg/dL), or hemoglobin A1c ≥6.5%.

^4^Hypertension will be defined by self-reported physician-diagnosed hypertension, use of antihypertensive medication, or systolic blood pressure ≥130 mm Hg or diastolic blood pressure ≥85 mm Hg.

^5^Chronic kidney disease will be defined by estimated glomerular filtration rate <60 mL/min/1.73 m² or an albumin-to-creatinine ratio >30 mg/g.
